# Promoting uptake of child HIV testing: an evaluation of the role of a home visiting program for orphans and vulnerable children in South Africa

**DOI:** 10.1080/09540121.2016.1176679

**Published:** 2016-07-08

**Authors:** Tonya R. Thurman, Brian Luckett, Tory Taylor, Melissa Carnay

**Affiliations:** ^a^ Highly Vulnerable Children Research Center, School of Social Work, Tulane University, New Orleans, LA, USA; ^b^ Department of Global Community Health and Behavioral Sciences, School of Public Health and Tropical Medicine, Tulane University, New Orleans, LA, USA

**Keywords:** HIV counseling and testing, orphans and vulnerable children, home visiting, South Africa, evaluation

## Abstract

HIV counseling and testing (HCT) is critical for children in generalized epidemic settings, but significant shortfalls in coverage persist, notably among orphans and others at disproportionate risk of infection. This study investigates the impact of a home visiting program in South Africa on orphaned and vulnerable children’s uptake of HCT. Using propensity score matching, survey data for children receiving home visits from trained community-based care workers were compared to data from children living in similar households that had not yet received home visits (*n* = 1324). Home visits by community-based care workers increased the odds of a child being tested by 97% (OR = 1.97, 95% CI = 1.34–2.92). The home visitation program had an especially pronounced effect on orphans, more than doubling their odds of being tested (OR = 2.12, 95% CI = 1.00–4.47) compared to orphans living in similar households that did not receive home visits. Orphan status alone had no effect on HCT independent of program exposure, suggesting that the program was uniquely able to increase testing in this subgroup. Results highlight the potential for increasing HCT access among children at high risk through targeted community-based initiatives.

## Introduction

The HIV continuum of care begins with timely diagnosis. Treatment can reduce HIV progression to AIDS by up to 75% (Violari et al., 2008), and early initiation of antiretroviral treatment (ART) is now universally recommended (Grinsztejn et al., 2014). Diagnosing the youngest patients is especially critical; without ART, more than half of infants and young children with HIV will die before reaching their second birthday (Newell et al., 2004). HIV counseling and testing (HCT) is also crucial for adolescents, among whom HIV-related mortality rose 50% between 2005 and 2012 – a period when HIV-related deaths overall fell by 30% (Joint United Nations Programme on HIV/AIDS [UNAIDS], 2013). HCT among adolescents may also help to prevent new infections by reinforcing prevention messages and encouraging safer sex practices (Olanike & Fawole, 2014; Rosenberg et al., 2013).

The World Health Organization calls for people of all ages to be tested in generalized epidemic settings; yet, access to and uptake of HCT by children is significantly lower than for adults (World Health Organization [WHO], 2013). Globally, an estimated 600 children under age 15 are infected each day (Joint United Nations Programme on HIV/AIDS [UNAIDS], 2014a). In South Africa, infection prevalence among children under age 15 may be as high as 6% in acutely affected areas (Ramirez-Avila et al., 2013). In 2012, UNAIDS estimated that 40% of new infections each day were among adolescents and youth aged 15–24 (UNAIDS, 2012). Orphans and vulnerable children (OVC) in particular are recognized as a priority population for testing in light of their elevated HIV risk from perinatal infection and greater sexual risk behavior (Newell et al., 2004; Operario, Underhill, Chuong, & Cluver, 2011; WHO, 2011, 2013).

Despite this greater risk, testing rates among OVC remain low. A study in South Africa among children accessing ART treatment at healthcare facilities found that orphans were likely to be diagnosed later than non-orphaned children (Mokgatle & Madiba, 2015). Another survey from South Africa found that fewer than half of 244 preschool children whose mothers were living with HIV had ever been tested (Chhagan et al., 2011). A recent national study similarly revealed that only 55% of children presenting at immunization posts whose mothers reported being HIV positive had their HIV status documented on their Road to Health Charts (Woldesenbet et al., 2015).

With support from the US President’s Emergency Plan for AIDS Relief, programs that serve OVC promote access to a package of key services including HCT (President’s Emergency Plan for AIDS Relief [PEPFAR], 2012). Rigorous evidence of these programs’ ability to achieve HCT service linkages, however, is rare (Institute of Medicine, 2013). One report from Zambia concluded that OVC home visiting programs increased the likelihood of testing among adult beneficiaries by 24% (Apicella, Schenk, & Khan, 2010), but studies of these programs’ effects on HCT among children are lacking. The present study responds to this gap by comparing HCT histories between children from households participating in a home visiting program versus matched nonparticipants in urban townships of South Africa.

## Methods

### Program model

Since 2001, Future Families has provided services to the families of OVC in three contiguous peri-urban townships (Mamelodi, Mamelodi East and Nellmapius) containing both formal and informal settlements northeast of Pretoria/Tshwane. The 2010 HIV rate among pregnant women in the greater Tshwane area was estimated at 26.1%: lower than the national prevalence rate, but among individuals in the lowest socioeconomic quintile, rates are expected to be much higher (Department of Health, 2011). Program services are targeted to households containing children who have lost one or both parents, chronically ill adults, and/or households in extreme poverty. Potential beneficiaries are visited in their homes by a care worker who collects detailed information about the family for eligibility determination. Households containing the most vulnerable children are prioritized for enrollment.

Under the supervision of a qualified social worker, care workers who have completed secondary education and are recruited from the community receive ongoing training from Future Families and make regular home visits to beneficiary households. A customized action plan is developed to meet the needs of each family, including material support, counseling, and referral for a variety of health and social services. Training for care workers includes an emphasis on HIV prevention and workers are encouraged to promote HIV testing and connect families to HCT providers.

### Study design and procedures

Children’s guardians were invited to take part in a standardized 30-minute oral interview in their homes during July 2014. Guardians were defined as the primary caregivers for the children in the household. Interviewers received fieldwork training from Tulane University’s Highly Vulnerable Children Research Center staff and were not affiliated with Future Families. This study takes advantage of a scale-up in Future Families programing to compare child HCT uptake among previous enrollees to that in new enrollees who had not yet received any services. All households who had been previously enrolled for approximately 18 months (*n *= 316) (receiving an average of 11 home visits over that time based on Future Families monitoring data), and those who were newly enrolled but had not yet received any services (*n *= 506) were invited to participate in the study. Of the total 822 households enrolled in the Future Families home visit program, 763 guardians from 763 households (93% response rate) completed baseline surveys: including 282 guardians who had been previously enrolled in the Future Families program (89% response rate) and 481 newly enrolled guardians (95% response rate) who had not yet received any services. Guardians provided information on every child under his/her care, including age, gender, whether each child’s parents are still living, and whether they had ever been tested for HIV. Guardians were also asked about their own demographic characteristics, HIV status, household assets and income and household composition.

While the program eligibility criteria were the same for both groups, previously enrolled households were generally more disadvantaged than newly enrolled households. Table 1 shows that previously enrolled households tended to have guardians with less education, more adults with a chronic illness (self-reported sickness for three months of the past year), be food insecure, have fewer assets and contain more orphans. We used propensity score matching to identify a subset of newly enrolled households most similar to those previously enrolled to create a quasi-experimental design.

**Table 1.** Household matching variables used to generate the propensity scores: before and after matching.

	Unmatched households					Matched households				
	Previous enrollees (*n *= 282)		New enrollees (*n *= 481)		Standardized difference	Previous enrollees (*n *= 231)		New enrollees (*n *= 231)		Standardized difference
Dichotomous variables	*n*	%	*n*	%	%	*n*	%	*n*	%	%
Male guardian	29	10.28	35	7.28	10.62	21	9.09	26	11.26	−7.18
Married guardian	135	47.87	245	50.94	−6.14	116	50.22	122	52.81	−5.18
Guardian without secondary education	125	44.33	132	27.44	35.77	91	39.39	81	35.06	8.97
Chronically ill guardian	66	23.40	73	15.18	20.95	44	19.05	42	18.18	2.24
Chronically ill other adult	97	34.40	120	24.95	20.80	68	29.44	65	28.14	2.87
HIV+ guardian	49	17.38	63	13.10	11.93	36	15.58	32	13.85	4.88
Guardian AIDS syptomatic^a^	27	9.57	28	5.82	14.11	18	7.79	19	8.23	−1.62
Guardian high daily functioning^b^	136	48.23	276	57.38	−18.41	120	51.95	117	50.65	2.60
Guardian has social support^c^	194	68.79	288	59.88	18.68	152	65.80	146	63.20	5.44
Guardian experienced stressful events^b^	256	90.78	418	86.90	12.35	209	90.48	210	90.91	−1.48
Guardian uses corporal punishment	158	56.03	249	51.77	8.55	124	53.68	137	59.31	−9.36
Food insecure household^d^	179	63.48	262	54.47	18.39	144	62.34	142	61.47	1.79
Informal settlement	76	26.95	214	44.49	−37.23	73	31.60	64	27.71	8.52
Earned income	116	41.13	200	41.58	−0.91	95	41.13	101	43.72	−5.24
Continuous variables	Mean	s.d.	Mean	s.d.	%	Mean	s.d.	Mean	s.d.	%
Guardian age	44.21	12.93	41.20	12.08	24.08	43.26	12.60	43.66	12.30	−3.16
Number of under 5s	0.79	0.88	0.73	0.79	7.94	0.77	0.85	0.74	0.83	3.61
Number of children 5–17	2.57	1.74	2.17	1.57	24.68	2.39	1.53	2.42	1.71	−1.60
Number of orphans	0.74	1.05	0.42	0.82	33.66	0.62	0.89	0.61	0.99	1.38
Number of adults	3.49	2.14	2.90	1.49	31.94	3.23	1.91	3.23	1.71	0.24
Number of HIV+ persons in home	0.05	0.22	0.02	0.17	12.68	0.05	0.22	0.04	0.22	5.94
Asset Index^e^	10.81	1.80	9.89	2.24	45.21	10.59	1.82	10.82	1.75	−12.84

^a^Cluver, Gardner, and Operario (2009); ^b^Self-generated; ^c^DeSilva et al. (2008);^d^Coates, Swindale, and Bilinsky (2007); ^e^Rutstein and Johnson (2004).

Propensity scores were calculated in SAS 9.3 software for each household using logistic regression predicting previous program participation. The propensity score models included 21 variables (as shown in Table 1) reflecting household vulnerability related to program participation eligibility including household and guardian demographic and socioeconomic factors. Matching was conducted using Coca-Perraillon’s (2007) greedy match macro without replacement and a caliper of 0.01. This method, designed to maximize the number of 1:1 matches while ensuring close similarity, resulted in 231 (82%) previously enrolled households matched to 231 (48%) newly enrolled households for an analytical dataset containing 462 households. Table 1 shows that after matching, households in both groups were similar with respect to the matching variables as indicated by the standardized differences.

Because the Future Families program operated at the guardian/household level, intervention and control status for each child was based on the program enrollment status (previous or new) of the household in which each child resided. Within the 462 households included in the analysis, there were 1324 children: 644 in the intervention households and 680 in control households. The analysis was performed using data for each child.

Approval to conduct this study was granted by the Tulane University Human Research Protection Program in the USA and the University of Limpopo Mendusa Research Ethics Committee in Pretoria South Africa prior to data collection. Informed consent was obtained from all participants.

### Analysis

This analysis examined whether or not a child had ever been tested for HIV, as reported by the caregiver. Multivariate logistic regression models were estimated using PROC SURVEYLOGISTIC in the SAS statistical software to adjust the standard errors for clustering of children by household. The predictor of primary interest was previous program participation, using an intent-to-treat design. The logistic regression models included basic demographics that may influence testing including children’s age, gender, and orphan status along with guardian’s age, gender, marital status, education, and whether the guardian was the child’s biological parent. All categorical variables were entered into the model as mutually exclusive dichotomous dummys. Additionally, a household asset index including 14 items based on the Measure DHS Wealth Index (Rutstein & Johnson, 2004) and household settlement type (formal or informal) were included in the model as socioeconomic proxy variables. Guardian’s knowledge of HIV transmission risk (as assessed with a series of five true/false questions) was included as a proxy for HIV awareness. A second model included the above variables as well as an interaction term for orphan status by program participation to test whether the program effect on HCT was dependent on being an orphan, given the higher risk for infection among this population.

Terms for the three townships were tested but eliminated because they were non-significant and did not improve model fit. None of the matched records contained missing data for any of the model terms.

## Results

Table 2 displays the background characteristics for the matched children by previous and new enrollee status (intervention and comparison group respectively) and bivariate results for any difference between the two groups. The analytical dataset contained similar numbers of male and female children, ranging in age from infancy to 17 years (median = 9 years). Over one-fifth of children in both groups had lost one or both parents, more than half were being cared for by a biological parent, and approximately 10% had an HIV positive biological parent. Only about half of the children had guardians who were married and about 10% had male guardians. While household characteristics were similar after matching, some characteristics of children in the two groups differed and these differences were controlled for in the analysis. Children in households that had previously participated in the home visit program more often had guardians who never attended secondary school, had guardians with lower HIV knowledge, and more often lived in informal settlements.

**Table 2.** Characteristics of children from matched households by program participation.

	Previous enrollees		New enrollees		
	(*n* = 680)		(*n* = 644)		*p*-Value
Dichotomous variables	*n*	%	*n*	%	
Male gender	328	48.24	318	49.38	0.6773
Orphan (single or double)	144	21.18	141	21.89	0.7507
Tested for HIV	301	44.26	192	29.81	<0.0001
Orphans tested for HIV	70	48.61	34	24.11	<0.0001
Children of HIV+ parent tested for HIV	49	64.47	37	60.66	0.6459
Male guardian	66	9.71	67	10.40	0.6729
Youth guardian (< 25 years)	15	2.21	25	3.88	0.0749
Married guardian	352	51.76	340	52.80	0.7075
Guardian without secondary education	224	32.94	176	27.33	0.0262
Guardian is not biological parent	282	41.47	247	38.35	0.2472
Guardian is HIV+ biological parent	76	11.18	61	9.47	0.3088
Guardian has correct HIV knowledge^a^	238	35.00	287	44.57	0.0004
Household in informal settlement	214	31.47	168	26.09	0.0307
Continuous Variables	Mean	s.d.	Mean	s.d.	
Age in years	8.51	4.96	8.49	4.89	0.9466
Household asset index^b^	10.60	1.79	10.83	1.71	0.0199

^a^Department of Health, South Africa (2007); ^b^Rutstein and Johnson (2004).

Forty-four percent of children from previously enrolled households had been tested for HIV, while 30% of children from newly enrolled households had been tested. The difference in testing was more pronounced for orphans, with 49% of orphans from previously enrolled households tested compared to 24% among children from newly enrolled households.

Table 3 presents results for logistic regression models predicting HIV testing in models both without and with an interaction term for orphan by previous participation (models 1 and 2, respectively). In both models, younger children were more likely to have been tested, with the odds of being tested falling by 6% for each additional year of life until age 17 (OR = 0.94, 95% CI = 0.91–0.96). The child’s gender was not associated with testing.

**Table 3.** Logistic regression predicting child HIV testing results both without (model 1) and with (model 2) an interaction term for orphans by program participation.

	Model 1				Model 2			
		95% CI		Log odds		95% CI		Log odds
	OR	Lower	Upper	*p*-Value	OR	Lower	Upper	*p*-Value
Age in years	0.94	0.91	0.96	<0.0001	0.94	0.91	0.96	< 0.0001
Male gender	1.07	0.83	1.38	0.6118	1.07	0.82	1.38	0.6278
Orphan (single or double)	1.16	0.79	1.72	0.4534	0.77	0.43	1.39	0.3869
Male guardian	0.32	0.14	0.72	0.0058	0.32	0.14	0.72	0.0063
Youth guardian (< 25 years)	0.18	0.04	0.69	0.0127	0.17	0.04	0.67	0.0115
Married guardian	1.05	0.71	1.55	0.8128	1.03	0.70	1.53	0.8816
Guardian without secondary education	0.96	0.61	1.50	0.8396	0.93	0.59	1.45	0.7364
Guardian is not biological parent	1.15	0.77	1.74	0.4934	1.15	0.77	1.73	0.5016
Guardian is HIV+ biological parent	3.14	1.77	5.56	<0.0001	3.15	1.79	5.55	< 0.0001
Guardian has correct HIV knowledge	1.70	1.13	2.54	0.0104	1.70	1.14	2.55	0.0100
Household asset index	1.06	0.93	1.20	0.4019	1.06	0.93	1.21	0.3615
Household in informal settlement	2.22	1.32	3.72	0.0026	2.22	1.33	3.73	0.0024
Enrolled previously (intervention group)	1.97	1.34	2.92	0.0007	1.69	1.09	2.62	0.0190
Enrolled previously* orphan	–	–	–	–	2.12	1.00	4.47	0.0494

In model 1, children with a male guardian had 68% lower odds than children with female guardians to have been tested (OR = 0.32, 95% CI = 0.14–0.72), while having a guardian younger than 25 years of age reduced a child’s odds of being tested by 82% (OR = 0.18, 95% CI = 0.04–0.69). Guardians’ education and marital status did not predict testing. Children cared for by someone who is not a biological parent had similar odds of being tested as children living with a biological parent. The odds that a child would be tested more than tripled for those whose guardian was a biological parent living with HIV (OR = 3.14, 95% CI = 1.77–5.56). Children living with a guardian who exhibited accurate knowledge of HIV transmission risks had 70% higher odds to be tested (OR = 1.70, 95% CI = 1.13–2.54). The household asset index was not predictive of child testing, but living in an informal settlement more than doubled the odds of a child being tested (OR = 2.22, 95% CI = 1.32–3.72).

Program participation (previous enrollees) increased the odds of a child being tested by 97% (model 1) (OR = 1.97, 95% CI = 1.34–2.92) compared to children from similar households that had not yet received services. When including an interaction term for orphan by program participation (model 2), orphans living in participating households had double the odds of being tested as non-orphans living in similar non-participating households. (OR = 2.12, 95% CI = 1.00–4.47). Orphan status alone was not significant (OR = 0.77, 95% CI = 0.43–1.39), although the conditional effect for program participation remained significant (OR = 1.69, 95% CI = 1.09–2.62).

## Discussion

HCT is an essential stepping stone to care and treatment, and the potential effects of successful programming to reach more children in generalized epidemic settings are profound. The implications for orphaned and vulnerable children, already at higher risk of HIV, are even more striking. The results of this study suggest that the Future Families home visiting program contributed to increased rates of HIV testing among beneficiary children relative to those living in similar circumstances who did not receive program services. Our findings demonstrate that orphans, who are at especially high risk for HIV due to mother-to-child transmission and higher rates of sexual risk behavior in adolescence, benefited from the home visitation program in particular. This research serves as a window into the factors associated with children’s access to HCT in one peri-urban setting in South Africa, as well as aspects of programming that may contribute to gains in testing prevalence.

Qualitative research from South Africa highlights a lack of money for transport and poor access to welfare grants as important factors influencing children’s access to HIV services (Kimani-Murage, Manderson, Norris, & Kahn, 2013). Poverty is typically compounded in households affected by HIV, the result of problems such as caregivers’ lost wages, increased medical and home care expenses, and a high child-to-adult ratio (South African Human Rights Commission & United Nations Children’s Fund, 2011). Orphans’ caregivers may also be unaware of the HIV status or testing history of a child for whom they have assumed responsibility, and may avoid testing due to fear of a positive diagnosis (WHO, 2013). Caregivers may also worry that a positive result will expose the child to stigma and/or unmask the caregiver’s own HIV-positive status – both already established as significant barriers to HCT in South Africa (Davies & Kalk, 2014; Ramirez-Avila et al., 2013). Children in HIV-affected households also experience high mobility, moving between homes as their caregivers become ill or pass away, another factor compromising access to health services (Foster & Williamson, 2000).

Prior research on OVC home visiting programs suggests that the one-on-one support, education and service referrals provided by care workers to beneficiary families have the potential to help mitigate some of these barriers to HCT. A longitudinal quasi-experimental program evaluation in South Africa demonstrated that paraprofessional home visiting services contributed to measurable gains in social grant uptake among OVC households, effectively increasing these families’ available income (Thurman, Kidman, & Taylor, 2015). The education and psychosocial support that home visitors provide may also help caregivers to understand the benefits of HIV testing, cope with the implications of a positive diagnosis and reduce stigma associated with HIV and AIDS. For example, a quasi-experimental program evaluation in Tanzania found that caregivers who received home visits had lower negative attitudes toward people affected by HIV, versus caregivers in a control group (Nyangara, Obiero, Kalungwa, & Thurman, 2009).

The Future Families home visiting program that is the focus of this study makes a concerted effort to promote HCT for children and other beneficiaries. The program incorporates an HCT-focused training module, refresher training, targeted encouragement from program staff and community resource information designed specifically to promote testing uptake. Similar results would undoubtedly be harder to achieve in home visiting programs without this special emphasis. In addition, the Future Families study took place in a peri-urban environment where service accessibility may be less constrained than in rural communities. However, recent national data from South Africa suggest that the disparity is slight, with 68% of people in urban areas reporting having accessed HCT services compared to 62% in rural communities (Shisana et al., 2014). Our use of an intent-to-treat design improves external validity of these results, suggesting that home visitation programs may be effective in other settings as well. Future research could help to identify the aspects of home visiting intervention models that are most influential for HCT uptake in different contexts, to help tailor programming.

This study has several important limitations. While propensity score matching is very effective in creating similar comparison groups, it does not necessarily address potential participation bias associated with needier households enlisting in the program earlier. Thus, the households in the intervention group may differ from those enrolled later with regard to factors not included in the analysis that could potentially affect HCT uptake. This quasi-experimental study establishes a strong correlation between program participation and child HIV testing, but cannot assign causality as with a truly experimental design. Furthermore, the questionnaire asked only if the child had ever been tested for HIV and not if the child had been tested since enrollment in the Future Families home visiting program. If child testing is associated with early enrollment in the Future Families home visits, the program effect may be inflated. Social desirability bias may also affect reports of HIV testing in the intervention group, given the program’s emphasis on education and prevention. Incorporating follow-up questions about the recency of testing and the factors that prompted it would provide useful additional information for better understanding HCT practices and distinguishing the program’s effects.

In spite of the encouraging results seen in this study, as many as 51% of orphans in the matched intervention group had never been tested for HIV. Programs serving OVC should consider ways to bolster HCT promotion. While there were too few examples in our data to establish clear associations, concerted efforts to reach children cared for by a male or youth may be particularly valuable, as children in these homes were far less likely to be tested. Higher testing prevalence among children with an HIV-positive guardian is encouraging. However, despite efforts to reduce mother-to-child transmission, pediatric testing is still considered a major challenge in South Africa (Meyers et al., 2007). Point-of-service issues affecting guardians’ proclivity to seek testing – such as long wait times, limited clinic hours, and communication or cultural barriers with HCT providers – may be difficult for home visitors to address. Some programs are beginning to offer home-based testing. A meta-analysis of 21 studies on home-based HCT in sub-Saharan Africa found an average acceptance rate of 83% (Sabapathy, Van den Bergh, Fidler, Hayes, & Ford, 2012). Future studies should investigate the cost effectiveness of home- versus facility-based testing in order to inform decision-making about optimal service delivery (WHO, 2013).

A clear imperative exists for innovative multi-sectorial strategies to increase HCT among children, especially in countries with generalized epidemics. Ensuring that 90% of people living with HIV know their status by 2020 (UNAIDS, 2014b) will require considerable coordinated effort, and civil society organizations have already been identified as important partners in the effort to expand HCT to those who need it most (Kellerman & Essajee, 2010; WHO, 2011). The results detailed here highlight the potential for community-based OVC programs to link high need, underserved groups to this critical lifesaving resource.
